# *MIR-107*, *MIR-223-3P* and *MIR-21-5P* Reveals Potential Biomarkers in Penile Cancer

**DOI:** 10.31557/APJCP.2020.21.2.391

**Published:** 2020

**Authors:** Jaqueline Diniz Pinho, Gyl Eanes Barros Silva, Antonio Augusto Lima Teixeira Júnior, Marta Regina de Castro Belfort, Juliana Melo Macedo, Isabela Wernerck da Cunha, Luciana Gonçalves Quintana, José de Ribamar Rodrigues Calixto, Leudivan Ribeiro Nogueira, Ronald Wagner Pereira Coelho, André Salim Khayat

**Affiliations:** 1 *Nucleus of Oncology Research, University Hospital João de Barros Barreto, *; 9 *Institute of Biological Sciences, Federal University of Pará, Belém, PA, *; 2 *Department of Pathology, Ribeirão Preto Medical of School, University of Sao Paulo, *; 5 *Antônio Prudente Foundation, Sao Paulo, *; 3 *Laboratory Immunofluorescence and Electron Microscopy, University Hospital Universitário Presidente Dutra, *; 4 *Laboratory of Genetics and Molecular Biology, Department of Biology, Federal University of Maranhão, *; 6 *Department of Medicine II, Federal University of Maranhão, *; 7 *Urologist at the Maranhense Institute of Oncology Aldenora Belo, *; 8 *Oncologist, Maranhense Institute of Oncology Aldenora Belo, São Luís, MA, Brazil. *

**Keywords:** miRNAs, prognosis, lymph node

## Abstract

**Background:**

Inguinal lymph node involvement is the main prognostic factor in patients with penile cancer. However, there is a lack of marker/s for lymph node metastasis. microRNAs have been investigated as potential markers for prognosis of various types of cancer. Taking this into consideration, our main goal was to determine the association of *miR-223-3p*, *miR-107*, and *miR-21-5p* expression with clinicopathological characteristics, as well as presence of lymph node metastasis in patients with penile cancer.

**Methods:**

Formalin-fixed paraffin-embedded penile squamous cell carcinoma specimens from 50 patients, at diagnosis and prior to any cancer treatment, were obtained. Tissue samples comprising at least 70% malignant cells and adjacent non-tumor tissues were evaluated by using qRT-PCR for expression level of *miR-223-3p*, *miR-107* and *miR-21-5p*. Additionally, molecular identification of HPV was performed by PCR, and the expression levels of *PTEN* were analyzed by immunohistochemistry.

**Results:**

Penile squamous cell carcinoma primary tumors presented higher expression of *miR-223-3p*, *miR-107*, and *miR-21-5p* when compared to non-tumor adjacent tissues. Upregulation of *miR-223-3p* was associated lymph node metastasis. Higher expression of *miR-107* was associated with worsening of prognosis (as observed by histological grade II and III, tumors bigger than 2.0 cm, stage III and IV, and lower disease-free survival). In addition, higher expression of miR-107 and *miR-21-5p* was correlated to the absence of PTEN protein expression.

**Conclusions:**

Our data demonstrate that higher expression of *miR-223-3p*, *miR-107*, and *miR-21-5p* is correlated with poor prognosis in penile cancer. The upregulation of these microRNAs potentially affect critical cancer pathways and may be important for the prognosis and response to therapy in penile cancer.

## Introduction

Penile cancer (PC) has a high incidence rate in developing countries such as Brazil (Coelho et al., 2018). The major risk factors for penile cancer are human papilloma virus infection and phimosis (Christoulidou et al., 2015). Moreover, owing to poor socio-economic factors, the diagnosis is delayed (Gao et al., 2016). Consequently, patients are usually diagnosed at an advanced stage, resulting in total or partial penectomy, and often present with regional lymph node metastasis and reduced survival (Protzel et al., 2009; Reis et al., 2011)

The factors of poor prognosis in patients with PC are: histological grade III and IV, advanced stage of tumor, vascular and perineural invasion, and lymph node involvement; the latter amongst these is considered the most relevant (Protzel et al., 2009). Treatment of PC involves lymphadenectomy, which is associated with post-surgical complications and high morbidity (O’Brien et al., 2017; Protzel et al., 2009). In addition, patients without diagnosable lymph nodes may present micro metastases (Leijte et al., 2007) and those with inguinal lymph node metastases have a disease-free survival rate of less than 40% for three years (Protzel et al., 2009). Therefore, prophylactically performed lymphadenectomy may confer morbidity to the individual. This necessitates the use of molecular markers for predicting inguinal lymph node metastases. 

Among the various molecular biomarkers, microRNAs have been considered potential prognostic markers of malignant neoplasm, since they may present differential expression profiles in different stage, types, regression grades, or differentiation states of cancer. In addition, they reveal new signaling pathways and therapeutic molecular targets, correlated with carcinogenesis (Weidan et al., 2017). In PC, the first large-scale study with microRNAs analysis was conducted by Zhang et al., (2015), and some differentially expressed microRNAs were identified, such as *miR-107*, and *miR-223-3p*. These micro RNAs were described related with metastasis, invasion, transport, and colonization in cancer (Gao et al., 2016; Hu et al., 2016; Markou et al., 2016; Mavrakis et al., 2011). Since PC is often accompanied with lymph node metastases, the involvement of these miRNAs in prognosis of this disease was checked. 

The present work aimed to analyze the expression of aforementioned miRNAs from patients exhibiting clinical-pathological characteristics of PC, with the ultimate goal of identifying potential biomarker/s that could aid in the understanding of this neoplasm and assist in determining therapeutic approaches.

## Materials and Methods

*Patients and sample collection*

A total of 50 formalin-fixed paraffin-embedded specimens of penile squamous cell carcinomas were included in this study. The samples were obtained from untreated patients who underwent tumor resection at Hospital Aldenora Belo and Hospital Presidente Dutra, located in the city São Luis, MA, Brazil, during 2013 to 2017. All tumors were classified according to the 8th edition of the American Joint Committee on Cancer (AJCC) (Amin, 2018). 

The samples were checked for areas of cancerous and non-malignant adjacent tissues using hematoxylin-eosin staining. Tissues comprising at least 70% malignant cells were included for further study. In patients with lymph node metastasis, a lymph node was selected and the matched adjacent non-tumor tissue 2 cm apart from the tumor site was selected. Subsequently, 10 micrometers of tumor from these selected areas were removed, stored in sterile tubes, and sent for RNA and DNA extraction.

The study was approved by the Scientific Commission - COMIC - HUUFMA with the opinion no. 2457/2014-60 and by the Research Ethics Committee of the University Hospital/CEP-HUUFMA with approval number: 1.093.435.

*HPV Molecular Identification*

For molecular detection of HPV, extraction of the genomic DNA from the samples was performed using the protocol of Sambrook et al. (1989). The nested PCR reactions were performed by using primers PGMY09 and PGMY11 for the first round, and primers GP5+ and GP6+ for the second round (Gravitt et al., 2000). These primers corresponds to the L1 region of the viral capsid. In addition, a pool of HPV 18-positive HeLa cells was used as a control.

HPV genotyping was performed for the samples identified as HPV-positive by the sequencing reaction with the DYEnamic ET Terminator Cycle Sequencing Kit, according to the protocol suggested by the manufacturer (GE Healthcare, Little Chalfont, UK).

*Follow up*

Routine follow-up included monthly physical examinations during the first year, every 6 months in the second, and annually from there on. Chest radiography was performed annually. The patients were classified into four categories at the end of follow-up: alive with cancer, alive without cancer, death from cancer, and lack of follow-up.

*Quantitative real time polymerase chain reaction (qRT-PCR)*

Total RNA was extracted using High Pure miRNA Isolation Kit (Roche Applied Science, UK).Reverse transcription reaction (RT) was performed with the MicroRNA TaqMan^®^ RT kit (Applied Biosystems) according to manufacturer’s instructions. The reaction mixtures were incubated at 16^o^C for 30 min, followed by 42^o^C for 30 min, then 85^o^C for 5 min before being held at 4^o^C. Quantitative PCR was performed on an ABI7500 fast real-time PCR system (Applied Biosystems). PCR cycling conditions included 50^o^C for 2 min and 95 ^o^C for 10 min, followed by 40 cycles of 95^o^C for 15 s and 60^o^C for 1 min. 

All reactions were performed in triplicate and normalized with an endogenous control (RNU6B). Data were analyzed by relative quantification using the comparative Ct method (Schmittgen et al., 2008).

The primer sequences used were: miR-223-3p (forward, 5’-ACACTCCAGCTGGGTGTCAGTTTGTCAAAT-3’ and reverse’-CTCAACTGGTGTCGTGGAGTCGGCAATTCAGTTGAGTGGGGTAT-3’); miR-107 (forward, 5′-ATACCGCTCGAGTGCCATGTGTCCACTGAAT-3′ 

and reverse, 5′- ATACCGCTCGAGTTCCATGCCTCAACTCCT-3′); miR-21-5p (forward, 5′- ACACTCCAGCTGGGTAGCTTATCAGACTGA-3’ and reverse 5’-TGGTGCGTGGAGTCG-3’); RNU6B (forward, 5′-CGC TTC GGC AGC ACA TAT AC-3′ and reverse 5′-TTC ACG AAT TTG CGT GTC AT-3′).

*Immunohistochemical analysis*

For the PTEN immunohistochemistry analysis, we used the primary monoclonal antibody EPR9941 (AbCam # ab154812) with a 1: 100 μc dilution rate and the scores were classified as described by Stankiewiecz et al., (2011) as: negative (score 0), low (score 1) and high expression (score 2). Sections of breast duct carcinoma were used as negative control.

*Statistical analysis*

Student’s t-test was used to compare microRNA expression levels with clinical-pathological parameters and protein expression. The ANOVA test was applied to compare the expression level of groups with more than two variables. The χ^2^ test was used to associate clinical-pathological and protein expression data. Survival analysis was performed using the Kaplan-Meier method to determine disease-free survival and overall survival compared with the level of microRNA expression. The log rank test was used to compare survival curves. All statistical analyses were performed using SPSS software (version 23.0, Chicago, IL, USA) and values with p ≤ 0.05 were considered statistically significant.

## Results

*Patient population*

Fifty patients with clinico-pathological diagnosis of penile squamous cell carcinoma participated in this study. The mean age of the patients was 62.3 years. There was a prevalence of patients with localized lesion in the glans (92.0%), histological grade II and III (88.4%), primary tumor pT2 and pT3 (80.0%), and tumor subtype usual (44.0%) (Table 1).

HPV infection was detected in 80.0% of the primary tumor samples. There was multiple infection in 30% (12/40) of the cases. The most frequent subtypes were HPV 16 (65.0%), followed by 59, 74, 73, 11, 30, 56, 58, 66, 6, 18, 44, 51, 53 and 63, no association was observed between HPV infection and the clinical-pathological variables (Table S1).

Regarding the surgical treatment, 70% (35/50) underwent partial penectomy, and 42% (21/50) underwent lymphadenectomy. Among the patients who underwent lymphadenectomy, 66.7% (14/21) were diagnosed with lymph node metastasis and 33.3% (7/21) had lymphadenitis. The average time interval between symptom onset and treatment was 20.8 months, and the mean follow-up was 17.3 months. During the follow-up period, five patients died from cancer, 41 stayed disease-free, and four discontinued follow-up. Among the 50 patients, recurrence of PC was observed in seven patients. Besides the normal treatment for PC, two patients were subjected to chemotherapy and seven to radiotherapy.

*microRNA expression profile in PC*

**Figure 1:**
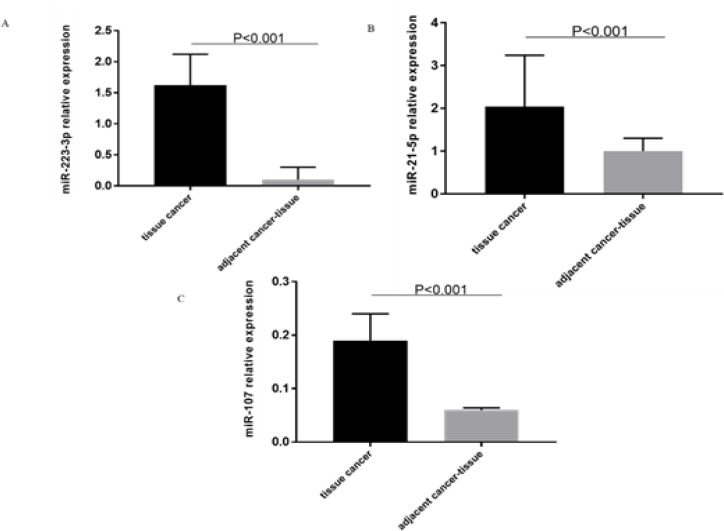
Relative Expression of MicroRNAs Comparing Primary Tumor Samples to Tissue Samples from Tumor Adjacent Region. A) Relative expression of miR-223-3p. B) Relative expression of miR-21-5p. C) Relative expression of miR-107

Expression levels of miR-223-3p, miR-21-5p, and miR-107 were evaluated in 50 paired samples cancerous and non-malignant adjacent tissues by RT-qPCR. As shown in Figure 1, the expression levels of miR-223-3p (fold change = 41.6; AUC= 0.80), miR-21-5p (fold change = 41.6; AUC= 0.80) and miR-107 (fold change = 64.3; AUC= 0.77) in the primary tumor were significantly (p < 0.001) higher than in non-tumoral adjacent tissue.

**Figure 2:**
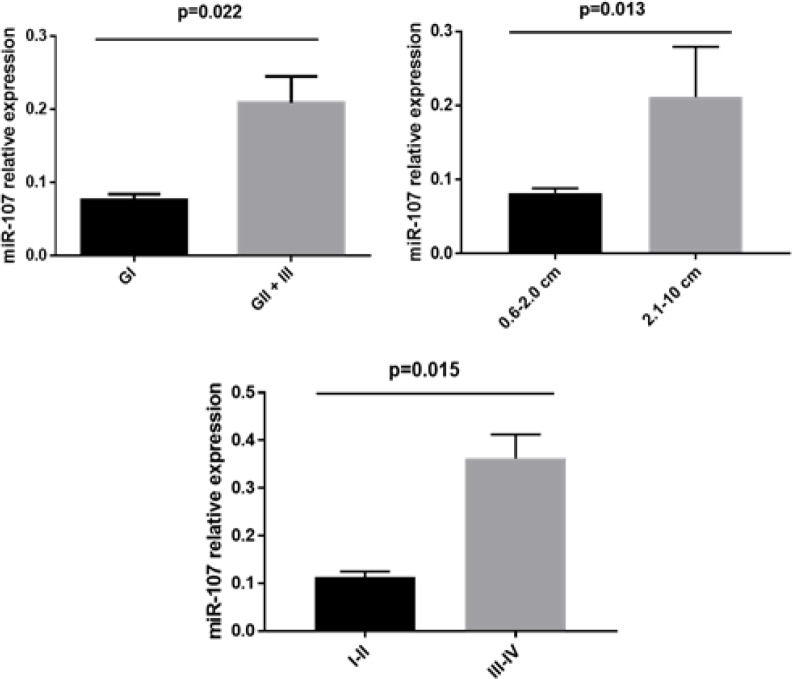
Relative Expression of miR-107 for Clinical-Pathological Characteristics. A) Relative expression of miR-107 for histological grade. B) Relative expression of miR-107 for tumor size. C) Relative expression of miR-107 for staging

A comparison of the clinical characteristics of PC with microRNA expression level revealed significant increase in miR-107 levels in patients with histological grade II and III (p = 0.022), tumors larger than 2.0 cm (p = 0.013) and staging III and IV (p = 0.015) Figure 2.

**Figure 3:**
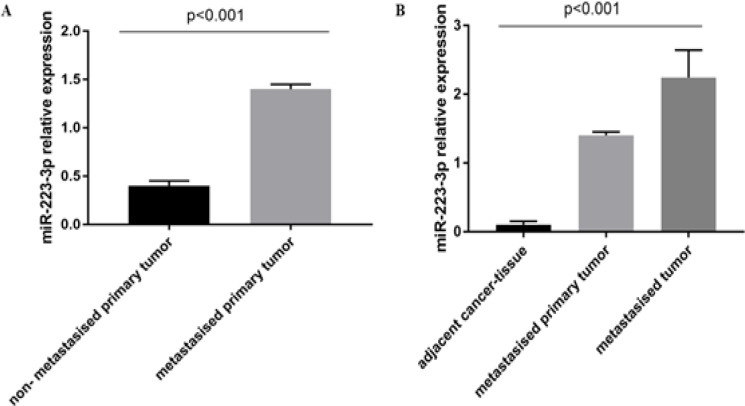
Relative Expression of miR-223-3p as to the Presence of Lymph Node Metastasis. A) Relative expression of miR-223-3p comparing primary tumor samples from patients with and without lymph node metastasis. B) Relative expression of miR-223-3p comparing tissue from the region adjacent to the tumor, primary tumor of affected patients with lymph node metastasis and metastatic tissue

The expression level of miR-223-3p was significantly (p < 0.001) higher in primary tumor samples from patients with lymph node metastasis when compared to that in non-tumoral tissue. Furthermore, significant (p < 0.001) expression of miR-223-3p was observed in samples of metastatic lymph nodes than in those of primary tumors from the same patient (Figure 3). Addicionally, no association was observed between HPV infection and the microRNAs analysed. 

**Figure 4:**
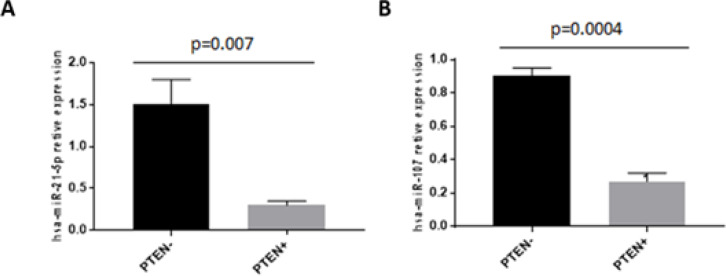
Relative Expression of miR-21-5p and miR-107 for the Absence of PTEN Protein Expression. A) Relative expression of miR-21-5p for absence of PTEN expression B) Relative expression of miR-107 for absence of PTEN expression

 Out of the 50 cases evaluated in this study, 43 were analyzed for PTEN protein expression. We observed loss of PTEN protein expression in 37 (86%) cases. This decrease in PTEN protein expression was statistically significant and correlated with high expression of miR-21-5p and miR-107 (Figure 4), no association was observed between PTEN protein expression and the clinical-pathological variables (Table S1).

*Survival analysis based on microRNA expression level*

**Figure 5:**
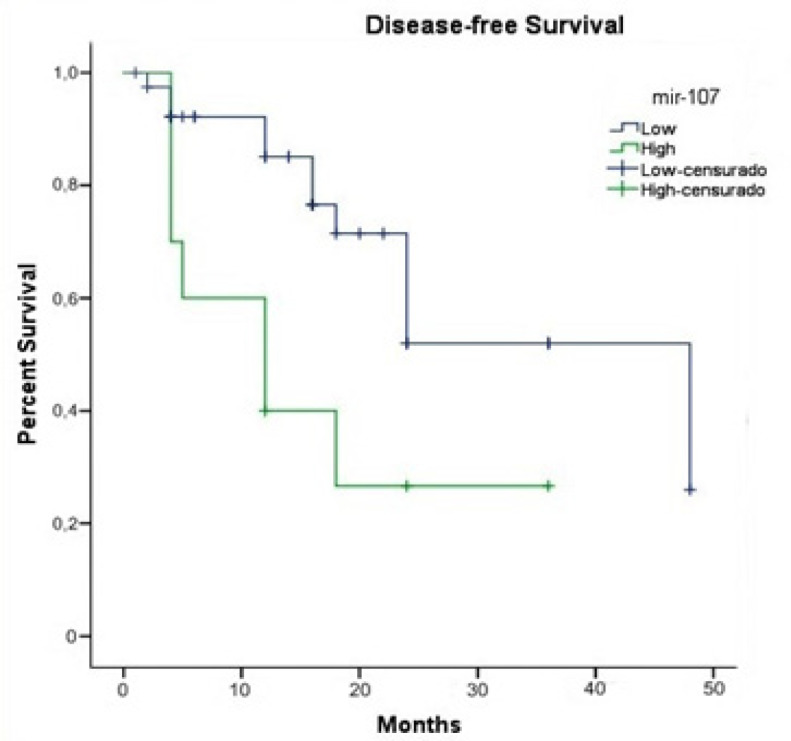
Patients with High miR-107 Expression have Reduced Disease-free Survival (log rank p = 0.042).

Our results showed that high miR-107 expression is associated with lower disease-free survival (log rank p = 0.042) (Figure 5). For the other microRNAs, no statistical difference was observed in relation to disease-free as well as overall survival.

**Table 1:** Clinical-pathological Characterization of Patients with Penile Cancer (N = 50).

Variables		N (%)
Tumor Subtype	Usual	22 (44.0)
	Warty	15 (30.0)
	Mixed*	9 (18.0)
	Basaloid	4 (8.0)
Tumor Size	0.6 - 2.0 cm	7 (14.0)
	2.1 – 5.0 cm	30 (60.0)
	≥ 5.1- 10 cm	13 (26.0)
Tumor Degree	1	6 (12.0)
	2	32 (64.0)
	3	12 (24.0)
Primary Tumor (pT)	pT1a	8 (16.0)
	pT1b	3 (6.0)
	pT2	15 (30.0)
	pT3	22 (44.0)
	pT4	1 (2.0)
	pTx	1 (2.0)
Perineural Invasion	Positive	19 (38.0)
	Negative	31 (62.0)
Lymphatic Invasion	Positive	15 (30.0)
	Negative	35 (70.0)
HPV	Positive	40 (80.0)
	Negative	10 (20.0)
PTEN (N=43)	Absence of expression.	37 (86.0)
	Expresso	6 (14.0)

* Mixed, 3 Usual and Basaloid; 6 Usual and Warty

## Discussion

In penile cancer, there is a scarcity of marker/s to diagnose lymph node metastasis, which hinders the decision to submit a patient to lymphadenectomy (Protzel et al., 2009). However, some molecular markers have been identified, that provide information on perineural (EGFR) recurrence and invasion (Silva Amâncio et al., 2017) epithelial-mesenchymal transition (E-cadherin and Vimentin) (Da Cunha et al., 2016) and lymph node metastasis (*miR-101*, *miR-1*, and *miR-204*) (Hartz et al., 2016).

The data presented in this work demonstrate that *miR-223-3p* is a potential biomarker of lymph node metastasis, because its overexpression was observed in samples of primary tumors from patients with lymph node metastasis when compared to expression in those who did not have metastatic tumors, in addition to higher expression in lymph node metastases samples compared to that in primary tumors from the same patients. These findings reinforce the potential influence of this microRNA on the process of lymph node metastasis, because its expression is higher in both groups. The expression of *miR-223-3p* is significantly higher in metastatic sites compared to primary tumors. This microRNA is known to be overexpressed in patients with colorectal cancer with lymph node metastasis or in advanced pathological metastatic disease (Li et al., 2012). Its high expression was also related to lymph node involvement in vulvar cancer, considered a female cancer with features similar to those of penile cancer (Maia et al., 2013).

Regardless of the correlation with lymph node involvement, we observed overexpression of *miR-223-3p *in squamous cell carcinoma. Previous studies have described this microRNA as an oncomir in penile cancer (Kuasne et al., 2017; Zhang et al., 2015). Munhoz et al., (2015) observed that *miR-223-3p* mediated low expression of *SLC8A1*, causing lower intracellular calcium concentrations, low rates of apoptosis, and increased proliferation of tumor cells in penile carcinoma. These studies illustrate the importance of this microRNA in carcinogenesis, especially in malignant neoplasm. Moreover, it has role in tumor initiation, progression, and metastasis process and is suggested to be a target candidate for cancer therapy (Gao et al., 2017).

In the present study, *miR-107* was highly expressed in tumors demonstrating an oncomir behavior, which corroborates a previous study performed using next generation sequencing and validated by qRT-PCR in penile cancer samples (Zhang et al., 2015). This is the first study that shows the association of this microRNA with worsening of prognosis in patients with penile cancer, displaying histological grade 2 and 3, tumors larger than 2.0 cm, and staging III and IV. In previous studies, overexpression of *mir-107* in colorectal and gastric cancers has been shown to suggest tumorigenic and metastatic potential (Kuasne et al., 2017; Xiong et al., 2017). Therefore, these data demonstrate the potential of miR-107 as a biomarker in the prognosis of penile cancer.

Low PTEN protein expression was related to high *miR-107* and* miR-21-5p* expression, indicating involvement of these miRNAs in the regulation of PTEN. In cervical cancer, miR-21-5p is a post-transcriptional regulator of PTEN, whose action decreases the gene and protein expression of PTEN (Peralta-Zaragoza et al., 2016). In colorectal cancer, high expression of *miR-21-5p *modulated malignant phenotypes, such as proliferation, anti-apoptosis, cell cycle progression, and cell invasion, through the regulation of PTEN protein expression (Wu et al., 2017).The target genes of the microRNAs discussed in this study regulate important pathways such as the mitogen-activated protein kinase pathway MAPK (ERK1/ ERK2),which when altered, can participate in the initiation and progression of cancer, and which have been recognized as important targets for therapy against various cancers (German et al., 2017). In addition, the *miR-223-3p *microRNA regulates the *FBXW7 *gene, which is a tumor suppressor, and is often mutated in several types of human cancers, including penile cancer in the advanced stage (Ali et al., 2016). 

In addition to the fact that these *miRNAs (miR-223-3p*, *miR-107,* and *miR-21-5p)* are predicted by a large number of genes that are active in the carcinogenic process, it is important to consider that they have their deregulation mediated by increased expression, which is necessary for therapeutic measures based on the reduction/inhibition of these, such as through the use of antagomir (Weidan et al., 2017). Furthermore, this study evidenced alterations in the expression of these microRNAs associated with relevant clinical and pathological characteristics as well as with molecular modifications in a tumor suppressor (PTEN) that is notably involved in the genesis of these tumors. Such epigenetic alterations can be useful when predicting lymph node invasion (main factor of worse prognosis in penile cancer) in preoperative biopsies or samples from the primary tumor destined to the pathology service, collaborating in the decision of the clinical-therapeutic conduction of the patients. Besides, these markers can be validated in non-invasive samples, such as plasma, serum and blood, since these microRNAs have already shown promising results in other types of cancers using samples of this nature (Kao et al., 2017; Komatsu et al., 2015).

In conclusions, the microRNAs discussed in this study are potential biomarkers for initiation of tumorigenesis, poor prognosis (miR-107), and lymph node involvement (miR-223-3p) in patients with penile cancer. To evaluate the clinical application of these findings, further studies are needed.

## Funding

The study was funded by the Fundação de Amparo à Pesquisa e Desenvolvimento Científico do Maranhão (FAPEMA) and Núcleo de Pesquisa em Oncologia/ HUJBB- UFPA. Those who provided funding have no role in study design, data collection, analysis, interpretation of data, writing of the paper, or the decision to submit the paper for publication. 
